# Supplementary material for: Multilevel negative binomial analysis of factors associated with numbers of antenatal care contacts in low and middle income countries: Findings from 59 nationally representative datasets
Source: PLoS One. 2024 Apr 18;19(4):e0301542. doi: 10.1371/journal.pone.0301542 (PMC11025891; doi:10.1371/journal.pone.0301542)
Supplement: Attachment — Submitted filename: general comments.docx [file pone.0301542.s001.docx]

Thank you so much for allowing me to revise the article.

**General comments**

1. line # 2 and 3 include sets. Instead of Findings from 56 nationally representative data. Better “Findings from 56 nationally representative data sets”.
2. Line #33# why you employed multilevel negative binomial regression, why not poison regression?
3. Why you focused only on individual variables? Why not include community level variables?
4. Line # 45# conclusions section: your recommendation is general beater specific
5. Line # 53# Keywords: Better focused on your title, what was Count modeling?
6. Line #67-69# re write the statement
7. Line #70-71# “Development Goals (SDGs) aimed to reduce maternal mortality (MMR) to 70 per 100,000 live births by 2030”. Please include globally
8. Line # 78# instead of still birth, better to say still births.
9. Line # 92 # is not clear, what does it mean “54% of maternal 95 deaths and 33% of stillbirths in LMICs”
10. Line # 140-141# is your data sets include all low and middle-income countries and what about the sample size is it weighted or un weighted?
11. Line #198-199# please briefly discuses the Intra-class Correlation Coefficient (ICC) and proportional change in variance (PCV)?
