# Supplementary material for: Multilevel negative binomial analysis of factors associated with numbers of antenatal care contacts in low and middle income countries: Findings from 59 nationally representative datasets
Source: PLoS One. 2024 Apr 18;19(4):e0301542. doi: 10.1371/journal.pone.0301542 (PMC11025891; doi:10.1371/journal.pone.0301542)
Supplement: Attachment — Submitted filename: point by point response R2.docx [file pone.0301542.s002.docx]

**Subject**: Point by point response for editor and reviewers

**Title:** Multilevel negative binomial analysis of factors associated with numbers of antenatal care contacts in low and middle income countries: Findings from 59 nationally representative datasets

**Manuscript ID:** PONE-D-22-24935R1

Dear editor and reviewers,

First and foremost we would like to express our gratitude for giving us those very critical and lesson-giving comments and suggestions to improve our paper. We have addressed all the reviewers' suggestions and comments to the manuscript. Our point-by-point responses for each comment and question are described in detail on the following pages. In addition, details of the changes are shown through Track Changes in the accompanying File.

**Comments and authors response**

**Journal Requirements:**

Please review your reference list to ensure that it is complete and correct. If you have cited papers that have been retracted, please include the rationale for doing so in the manuscript text or remove these references and replace them with relevant current references. Any changes to the reference list should be mentioned in the rebuttal letter that accompanies your revised manuscript. If you need to cite a retracted article, indicate the article’s retracted status in the References list and also include a citation and full reference for the retraction notice.

**Response:** Thank you. We have revised completeness and correctness of references accordingly. For example, references 7, 9, 11, 18, 23, and others references completeness and correctness were checked.

Explain clearly why only 56 countries were included and why others like India were left out. The justification that only countries with DHS data between 2010 and 2020 were included do not hold as countries like India and Sri Lanka had surveys within this time-period. Please explain why these and other such countries were left out. Also arrange Table-1 in alphabetical order of the name of the countries

**Response:** Thank you for your concern: We have included India and other two countries (Madagascar and Mauritania) which were left out previously and update dataset for Burkina Faso, Nepal, Cambodia Cote d’vore, Philippines, Tanzania, and Kenya in the revised manuscript. We are aware of Sri Lanka has 2016 demographic and health survey, however, we haven’t found Sri Lanka’s 2016 demographic and health survey at [archive@dhsprogram.com](mailto:archive@dhsprogram.com) website.

Please edit the following sentence “A total of 912,062 reproductive-age (15-49 years) were interviewed in 56 LMICs.” Do you mean women of reproductive age group?
**Response:** Thank you. We have revised it (methods, page 6, line 122-23).
Please edit the following sentence “Media exposure was generated from women’s asked on frequency of radio, ….”. What do you mean by “women’s asked on”?

**Response:** Thank you. We have revised it accordingly (methods, page 8, line 147-49).
In this sentence, do you mean ‘not a big problem’ (if a woman didn’t report none of the above problem were considered as no a big problem)?
**Response:** Thank you. We have revised it (methods, page 8, line 162-63).
You do not need to write ‘times’ after the % in the following sentences –
big problem had 12% times lower ANC contacts
ANC after 12 weeks of gestation had 18% (IRR=0.82, 95% CI: 0.81, 0.83) times lower ANC contacts
**Response:** Thank you. We have deleted times after the %.
There is a need to carefully edit out all grammatical typing errors in the manuscript. There are a lot of them.

**Response:** Thank you. We have carefully revised grammatical errors in the revised manuscript.

**Reviewer #2:**

The researcher must point out the Sustainable Development Goals and maternal mortality

**Response:** Thank you. The Sustainable Development Goals and maternal mortality are described in background section (page 3, line 56-64).
The researchers did not mention the reference they relied on to apply the methodology or the package used in STATA program

**Response:** Thank you. We have provided references accordingly.
The researchers did not mention the details of the model used, the form of the equation, and its statistical assumptions

**Response:** Thank you. We have provided details of the model used, the form of the equation, and its statistical assumptions in the revised manuscript (methods, page 9-11, line 176-220).
In presenting the results, researchers should focus on commenting on percentages and not mention both numbers and percentages at the same time in sentences.

**Response:** Thank you. Only percentages are used to describe respondents’ characteristics (results, page 12&13, line 252-58).
The researchers overlooked an important variable, which is the number of previous children the woman had given birth to. The more children a woman has, the less likely she is to receive prenatal care during pregnancy, as the woman becomes more experienced and less afraid of pregnancy. Why did researchers exclude this variable?

**Response:** Thank you. We included birth order in previous analyses, which is similar to parity (the number of children a woman has had previously) in our case (study population). However, to make more clearer to readers and to use meaningful categories, we have replaced birth order with parity in the revised manuscript.
At the head of Table 4, researchers should differentiate between IRR and AIRR, You must explain the difference between them **Response:** Thank you. IRR indicates crude incidence rate ratio and AIRR indicates adjusted incidence rate ratio. To assess factors associated with the number of ANCs, we used the AIRR, an epidemiologically relevant effect measure that adjusts for all important confounders. Sorry for our previous mistake, we have now replaced IRR with AIRR.

Researchers should avoid large paragraphs in the discussion, and the discussion section should be shortened because it is very long

**Response:** Thank you. We have revised accordingly (one paragraph includes single idea).

Reviewer #3:

1. Key words should be precise, not a combination of four or five word.

**Response:** Thank you. We have revised key words in the revised manuscript.

2. In abstract part write some finding in words, do not use numbers always.

**Response:** Thank you. We have used words to explain our findings in abstract section (page 2, line 37-42).
3. The policy suggestion in abstract is not particular at all, authors need to specify who should be the authority for policy interventions.

**Response:** Thank you. Now we revised it (page 2, line 44-48).
4. Why the study undertakes 56 countries for the study? Yes, I understand it is for LMIC’s. But who should be responsible for policy implications? As countries are located at different continents. Please write rationale behind the study in one or two sentence.

**Response:** Thank you. We conducted this study in low- and middle-income countries to increase the power of the study because, as shown in the background section, there are conflicting findings regarding factors in antenatal care. Low and middle income countries use similar WHO guidelines for antenatal care. Therefore, local stakeholders and international stakeholders such as the World Health Organization should take responsibility for antenatal care.
5. In discussion part many of the articles used as “Previous studies”, are studies conducted in India and some articles from developed countries. But, the 56 countries for the study, either does not contain India, nor there is ant developed country undertaken.

**Response:** Thank you. We have included India in the revised manuscript.
6. I suggest to remove such studies as the economic, political, social, and cultural setup are varies across nations and regions.

**Response:** Thank you. We have included India in the revised manuscript and only one reference from developed countries have been used to support general facts.
